# Engineering immune-competent hair follicle microphysiological systems: from organoid assembly to dynamic immune modelling

**DOI:** 10.3389/fbioe.2026.1846566

**Published:** 2026-06-08

**Authors:** Hanxiao Qu, Liudong Tong, Qinyi Lou, Lijun Qing, Maocan Tao, Hongbin Luo

**Affiliations:** 1 Zhejiang Chinese Medical University, Hangzhou, Zhejiang, China; 2 The First Affiliated Hospital of Zhejiang Chinese Medical University (Zhejiang Provincial Hospital of Chinese Medicine), Hangzhou, Zhejiang, China

**Keywords:** disease modelling, hair follicle organoids, hair regeneration, immune microenvironment, microphysiological systems

## Abstract

Hair follicle organoids (HF organoids) have progressed from simple epithelial–mesenchymal aggregates to spatially organized follicle-like structures and skin–hair follicle composite models. However, most current systems still focus on follicular morphogenesis and structural reconstruction, with limited capacity to recapitulate the immune, vascular, neural, and dynamic regulatory features of the native hair follicle (HF) microenvironment. This limitation is particularly relevant to immune-mediated hair disorders, such as alopecia areata, lichen planopilaris, and frontal fibrosing alopecia, in which immune privilege (IP) collapse, inflammatory infiltration, hair follicle stem cell (HFSC) niche damage, and tissue remodelling are central pathological events. In this Review, we discuss the transition from structurally oriented HF organoids towards immune-competent hair follicle microphysiological systems (HF-MPS). We summarize the structural basis of HF engineering, define immune competence as a functional state involving IP maintenance, immune-cell regulation of HF cycling and regeneration, and controllable inflammatory injury, and examine engineering strategies including local immune microenvironment control, compartmentalized culture, dynamic perfusion, vascularized and innervated interfaces, and quantitative monitoring. We further discuss applications in disease modelling, dynamic drug evaluation, patient-derived platforms, and regenerative medicine, while highlighting evidence boundaries and technical limitations. Immune-competent HF-MPS should be viewed as evolving human-relevant platforms rather than mature replacements for existing models.

## 1 Introduction

The hair follicle (HF) is a dynamic mini-organ that undergoes cyclic transitions through anagen, catagen, telogen, and regeneration. These cyclic transitions are orchestrated by epithelial-mesenchymal interactions (EMI), inductive cues from dermal papilla cells (DPCs), the hair follicle stem cells (HFSC) niche, the extracellular matrix (ECM), and the coordinated activities of local neural, vascular, and immune components (Stenn and Paus, 2001; Schneider et al., 2009; Lee and Choi, 2024). As an epithelial-mesenchymal skin appendage, the HF also maintains a specialized local immune status, including immune privilege (IP) in the lower anagen follicle and an immune-protective microenvironment surrounding the HFSC niche (Ito, 2010; Bertolini et al., 2020; Tan and Chang, 2024). These biological features make the HF not only a powerful model for studying epithelial regeneration and stem cell regulation, but also a unique system for interrogating immune tolerance, inflammatory injury, and tissue remodeling.

Advances in tissue engineering and organoid technologies have enabled substantial progress in the *in vitro* construction of HF-like structures. Hair follicle germs (HFGs), HF organoids, and skin organoids containing HF-like appendages have each advanced HF reconstruction from distinct yet complementary perspectives: early follicle-forming units, three-dimensional self-organizing systems, and integrated skin–HF composite models, respectively (Nakao et al., 2007; Lee et al., 2020; Hong et al., 2023). In parallel, biomaterial-based and bioengineering strategies, including ECM hydrogels, microsphere carriers, and three-dimensional assembly approaches, have improved cell adhesion, localized signal presentation, and spatial patterning, thereby enabling more controlled formation of HF-like tissues (Higgins et al., 2013; Kageyama et al., 2022; Zhang et al., 2026). Together, these developments have moved HF organoids beyond simple cellular aggregation toward more refined spatial organization and niche recapitulation.

Nevertheless, most existing models still prioritize follicle morphogenesis and structural recapitulation, focusing on HFG assembly, HF-like architecture, and hair shaft-like structure formation. By contrast, key dimensions of HF immune physiology remain insufficiently incorporated, including immune-cell input, local inflammatory perturbation, dynamic perfusion, vascularized interfaces, and real-time monitoring of structural, metabolic, and immune responses (Abreu and Marques, 2022). This limitation becomes particularly consequential in the context of immune-mediated HF diseases. Under physiological conditions, the HF relies on local immune homeostasis to maintain IP, preserve the HFSC niche, and sustain cyclic regeneration. In diseases such as alopecia areata (AA), lichen planopilaris (LPP), and frontal fibrosing alopecia (FFA), immune dysregulation can lead to IP collapse, immune-cell infiltration, HFSC niche damage, and inflammatory remodeling (Philpott et al., 1996; Bertolini et al., 2020; Senna et al., 2022). Thus, immune regulation represents a central axis connecting physiological homeostasis, injury repair, and disease-associated tissue destruction. Static epithelial-mesenchymal organoids are therefore insufficient to capture these dynamic immune-tissue interactions. HF models must accordingly advance from structurally oriented HF organoids toward immune-competent hair follicle microphysiological systems (HF-MPS) that can integrate local immune regulation, inflammatory perturbation, dynamic perfusion, and tissue repair responses.

Immune competence should not be conflated with the mere addition of immune cells to HF organoids. The central challenge is to maintain multiple cell types in relatively stable and functionally relevant states within the same system over extended culture periods. Epithelial cells, DPCs, HFSCs, immune cells, endothelial cells, and neurons differ substantially in their culture requirements, metabolic dependencies, and *in vitro* survival profiles. As construct size increases, limitations in oxygen and nutrient diffusion, metabolic waste accumulation, and local hypoxia become further exacerbated (Wörsdörfer and Ergün, 2021; Zhao et al., 2021). Meanwhile, the formation and maturation of HF-like structures generally require prolonged culture, whereas primary immune cells are prone to apoptosis, exhaustion, or phenotypic drift. These temporal and biological mismatches make conventional static co-culture systems poorly suited to the stable modeling of HF immune processes (Sun et al., 2019; Papp et al., 2024). Even when perfusion systems are introduced, fluid shear stress must be carefully controlled to avoid nonphysiological disruption of epithelial architecture and the HFSC niche (Nahon et al., 2024). Thus, HF-MPS should not simply adopt a generic MPS configuration; rather, they need to be specifically designed and validated according to HF architecture, cyclic behavior, immune status, and niche requirements. As non-animal testing approaches receive increasing attention, human-relevant MPS platforms are gaining research and translational value. However, their defining criterion should remain biological relevance, not the mere accumulation of engineering complexity (Carratt et al., 2025).

This review examines how HF organoid models can progress from structural reconstruction toward immune-competent HF-MPS. We begin by outlining the structural foundations of HF modeling, including HFG assembly, EMI reconstruction, ECM-mediated niche regulation, and skin-HF composite construction. We then define the functional basis of HF immune competence, focusing on IP maintenance, immune involvement in hair cycling and regeneration, and inflammatory damage following immune dysregulation. On this basis, we discuss engineering strategies for immune microenvironment control, compartmentalized culture, air-liquid interface (ALI) establishment, dynamic perfusion, vascularized and innervated interfaces, and quantitative monitoring. We further consider potential applications in disease modeling, dynamic drug evaluation, patient-derived systems, and regenerative medicine, while clarifying the current limits of evidence. To avoid conceptual ambiguity, we use HFGs, HF organoids, skin–HF composite models, HF-MPS, and immune-competent HF-MPS according to the working definitions provided in Table 1.

**TABLE 1:** Key concepts and working definitions in hair follicle organoid and MPS engineering.

Hair follicle germs (HFGs)
HFGs are early hair follicle primordium-like structures generated by the high-density recombination, self-organization, or engineered spatial arrangement of epithelial and mesenchymal cells, particularly dermal papilla cells (DPCs). They are mainly used to recapitulate epithelial–mesenchymal interactions (EMI), initiate folliculogenesis-related signaling, and study DPC inductive capacity. However, HFGs do not fully reproduce the native follicle microenvironment and are limited in modeling immune privilege (IP), vascular recruitment, neural regulation, and long-term cyclic transitions.

Hair follicle organoids (HF organoids)
HF organoids are three-dimensional (3D) follicle-like structures derived from stem cells, DPCs, keratinocytes, or pluripotent stem cell-derived cells. They emphasize 3D self-organization, extracellular matrix (ECM) support, follicle-like compartmentalization, and hair shaft-like or skin appendage-like structure formation. Although some can be integrated into epidermis–dermis-like architectures, most remain structurally oriented and lack stable dynamic perfusion, immune-cell recruitment, IP maintenance, and long-term cyclic regulation.

Hair follicle microphysiological systems (HF-MPS)
HF-MPS are engineered *in vitro* platforms that integrate follicle-like structures with modules such as compartmentalized culture, air–liquid interface (ALI), dynamic perfusion, vascularized interfaces, sensors, and quantitative readouts. Their defining feature is a dynamic tissue-like microenvironment, rather than structural complexity alone. Without functional immune-cell input, immunoregulatory signaling, and validated inflammatory responses, HF-MPS should be regarded as dynamically engineered follicle models rather than immune-competent systems.

Immune-competent HF-MPS
Immune-competent HF-MPS are HF-MPS platforms with validated immunoregulatory function. They should go beyond the detection of immune cells or immune markers and meet at least three criteria: maintenance of local immune tolerance and IP under homeostatic conditions; immune-cell regulation of HFSC activation, hair cycle transitions, and post-injury regeneration; and measurable inflammatory responses, such as IFN-γ/JAK–STAT activation, effector T Cell or myeloid-cell involvement, HFSC niche damage, and recovery after pharmacological or immunomodulatory intervention.

## 2 Structural foundations for engineering hair follicle organoids

**FIGURE 1:**
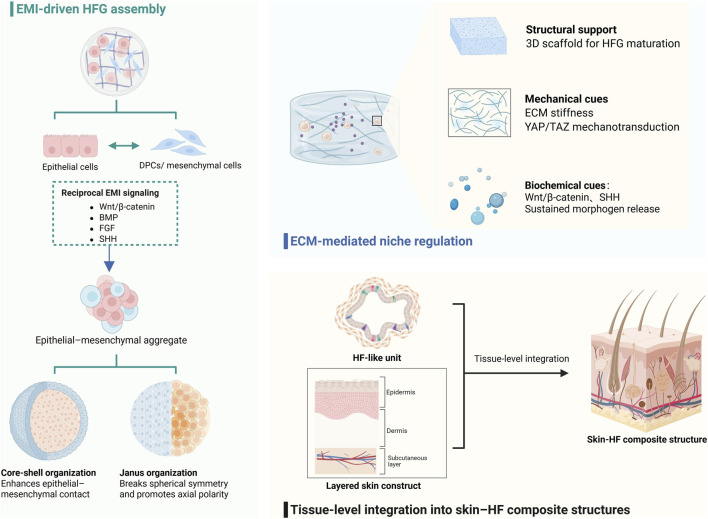
Structural foundations for engineering HF organoids: from EMI-driven HFG assembly to skin–HF composite structures.

The *in vitro* construction of HF organoids begins with the reconstruction of follicular architecture. As a highly specialized skin appendage, the HF does not form through simple cell aggregation; rather, HF-like structure formation requires inductive EMI, controllable HFG spatial organization, an appropriate ECM niche, and integration with stratified skin tissue (Lin et al., 2022; Park, 2022). Figure 1 summarizes this structural trajectory, from EMI-driven epithelial–mesenchymal aggregation and HFG spatial organization to ECM-mediated niche regulation and tissue-level integration into skin–HF composite structures.

### 2.1 Epithelial-mesenchymal interaction: inductive signaling for hair follicle germ assembly

EMI represents the cardinal inductive mechanism governing hair follicle morphogenesis and HFG assembly. Epithelial cells require mesenchymal cues to undergo growth, differentiation, and HF-like tissue formation (Kageyama et al., 2022; Mao et al., 2022; Hamida et al., 2024). Within this process, DPCs serve as the key mesenchymal component, regulating HFG formation, hair shaft differentiation, and subsequent cyclic regeneration through pathways such as Wnt/β-catenin, BMP, SHH, and FGF (Mao et al., 2022). Accordingly, the first challenge in HF organoid construction is to reconstitute inductive EMI between epithelial cells and DPCs *in vitro*.

Existing studies show that EMI can be reconstructed *in vitro*, but its effectiveness depends on spatial organization and preservation of DPC inductive capacity. Even dissociated epithelial and mesenchymal cells can re-establish EMI when reassembled at high density within a compartmentalized interface (Nakao et al., 2007). Higgins and colleagues further showed that DPCs expanded in two-dimensional culture readily lose their hair-inductive capacity, whereas three-dimensional culture, such as hanging-drop or hyaluronic acid-based systems, can restore their ability to induce *de novo* HF formation (Higgins et al., 2013). These findings are further supported by human DPC–keratinocyte models showing that spatial assembly modes shape HF-like structure formation (Kalabusheva et al., 2017). Together, these studies indicate that EMI reconstruction is not a simple co-culture, but a coordinated process requiring both DPC inductive-state maintenance and three-dimensional epithelial-mesenchymal spatial organization. Improving the uniformity and reproducibility of HFGs, therefore, requires a shift from cell composition alone toward rational spatial design.

### 2.2 Spatial organization: core-shell and janus configurations of hair follicle germs

As a skin appendage with defined growth directionality and spatial compartmentalization, the HF requires not only epithelial-mesenchymal contact but also the precise spatial arrangement of epithelial cells and DPCs within HFGs. Although conventional spherical aggregates provide three-dimensional cell-cell contact, they insufficiently recapitulate the directional organization and regional patterning of HF development. Spatial-configuration design has therefore become an important strategy for shifting HFG construction from random cellular aggregation toward more organized structural reconstruction.

Core-shell organization refers to a defined inner-outer architecture in which epithelial cells preferentially occupy the central region, whereas mesenchymal cells are distributed in the outer compartment. By modulating the three-dimensional microenvironment, particularly Matrigel concentration, epithelial and mesenchymal cells can self-organize into core-shell HFG structures, achieving nearly 100% functional HF induction *in vitro* and generating elongated hair shaft-like structures (Kageyama et al., 2022). This architecture concentrates and orders the epithelial-mesenchymal interface, thereby providing a favorable spatial context for early EMI and HF induction.

However, core-shell organization remains a largely spherical structure based on inner-outer compartmentalization, whereas the HF is an asymmetric organ with pronounced axial polarity. To break this initial spherical symmetry, Janus structures, which are anisotropic physical entities with two distinct sides, have been introduced into HFG engineering (Marschelke et al., 2020). Janus collagen microbeads fabricated by microfluidics can position epithelial and mesenchymal cells in opposite hemispheres to form side-by-side HFG-like structures. This asymmetric design enables functional HFG formation and improves *in vivo* hair-generation efficiency by more than twofold compared with conventional symmetric structure (Sugiyama et al., 2024). This strategy differs from the spontaneous polarization observed in long-term cultures derived from induced pluripotent stem cells (iPSCs) (Lee et al., 2020). Rather than waiting for polarity to emerge, the Janus design presets epithelial and mesenchymal positioning during early assembly, thereby generating more controllable asymmetric HFG organization.

Thus, core-shell and Janus designs should not be viewed as merely parallel fabrication approaches. The former optimizes the epithelial–mesenchymal contact interface, whereas the latter addresses asymmetric compartmentalization and directional organization. Compared with conventional three-dimensional co-aggregation, these strategies improve not only cellular contact but also the efficiency and reproducibility of HFG construction through controllable spatial organization.

### 2.3 Extracellular matrix-mediated niche regulation: structural, mechanical, and biochemical support

In HFG construction, EMI provides the core inductive mechanism, whereas core–shell and Janus architectures improve the order and controllability of epithelial–mesenchymal contact by regulating the initial spatial arrangement of cells. However, HFGs are not isolated cellular aggregates. Their further maturation requires sustained support from the surrounding ECM niche. Under physiological conditions, the ECM helps maintain DPC hair-inductive capacity and regulate HFSC cycling through matrix stiffness, molecular composition, and the storage and release of local morphogenetic signals (Zhou et al., 2021; Cheng et al., 2023; Di et al., 2023; Tan et al., 2025). Reconstructing an ECM microenvironment that approximates the native HF niche is therefore essential for advancing HFG-like aggregates toward HF-like units.

ECM-mimetic materials are designed to recapitulate the composition and mechanical features of the dermal matrix, providing epithelial cells and DPCs with a three-dimensional environment that supports adhesion, aggregation, and HFG-like structure formation (Gan et al., 2023). Commonly used matrices include natural or naturally derived materials, such as collagen, gelatin, hyaluronic acid (HA), and glycosaminoglycans (GAGs), as well as engineered hydrogels (Fernández-Martos et al., 2019; Kang et al., 2022; Li et al., 2024). For example, gelatin/HA hydrogel bioinks have been used to construct skin equivalents containing epidermal, papillary, dermal, and HF-like structures, indicating that naturally derived hydrogel composites can support stratified skin architecture and HF-like unit formation (Kang et al., 2022). GAG-based hydrogels can also mimic the dermal matrix, support sustained *ex vivo* growth and survival of human HFs, and are associated with activation of Wnt/β-catenin-related signaling (Fernández-Martos et al., 2019). These findings suggest that ECM-mimetic materials provide not only three-dimensional structural support, but also a dermal-like biochemical milieu that contributes to HF growth and structural maintenance.

Beyond composition, ECM mechanics also shape cellular behavior and niche function. Matrix stiffness, fiber alignment, and local mechanical properties can regulate cell fate through mechanotransduction pathways, with YAP/TAZ signaling serving as a key mechanism by which cells sense ECM mechanical cues (Cheng et al., 2023; Di et al., 2023). This is particularly relevant to HF organoid engineering because DPCs readily lose hair-inductive capacity during conventional two-dimensional expansion, whereas three-dimensional matrix environments can partly restore or preserve their inductive features (Higgins et al., 2013). Thus, optimizing ECM mechanical parameters is not merely a matter of material tuning, but a prerequisite for maintaining DPC inductive states, stabilizing the HFSC niche, and supporting continued maturation of HFG-like structures.

The ECM can also function as a local delivery platform for morphogenetic signals and growth factors. Strategies such as layer-by-layer (LbL) self-assembly, nanoscale ECM construction, and biphasic hydrogels have enabled sustained release of BMP2, Wnt3a, and related cues *in vitro*. These approaches can regulate HFSC quiescence-activation transitions, activate HF induction-related pathways, and promote functional HFG formation (Chen et al., 2021; 2024).

Accordingly, ECM-mediated niche regulation should be regarded as an essential foundation for advancing structurally oriented HF organoids toward functional HF-MPS.

### 2.4 Tissue-level integration: assembly of skin-hair follicle composite structures

As a skin appendage, the HF is embedded within a tissue environment composed of the epidermis, dermis, and subcutaneous tissue. Its morphogenesis, hair shaft outgrowth, and interaction with the surrounding matrix are shaped by the architecture of adjacent skin compartments. After epithelial–mesenchymal assembly, HFG spatial organization, and ECM-mediated niche regulation have been established, structural engineering must further integrate HF-like units into epidermal–dermal architectures to generate skin–HF composite structures that more closely approximate native skin organization.

Human pluripotent stem cell-derived skin organoids provide one route for generating complex appendage-containing skin models. Such systems can contain stratified epidermis, HFs, sebaceous glands, neurons, and other skin-associated components (Lee et al., 2020). However, their prolonged culture duration and complex induction procedures may limit efficiency, reproducibility, and broader application. In parallel, tissue engineering strategies, particularly three-dimensional bioprinting and micromolding, offer more controllable approaches for assembling stratified skin constructs with HF-like units.

Gelatin/HA hydrogel bioinks, for example, have been used to construct skin equivalents containing epidermal, papillary, dermal, and HF-like structures, illustrating that biomimetic hydrogel-based printing can support the spatial organization of stratified skin layers and appendage-like units (Kang et al., 2022). Similarly, three-dimensional printed molds can be used to pre-pattern microwells within collagen gels, creating defined dermal niches for DPC seeding and subsequent keratinocyte organization into HF-like units with dermal papilla, hair sheath, and inner root sheath-like features (Abaci et al., 2018). These examples indicate that skin-HF composite structures can be assembled through tissue-level engineering strategies, including bioprinting, micromolding, pre-patterned dermal niches, and the embedding of HF-like units into stratified skin constructs.

Thus, the transition from HFGs to skin–HF composite structures reflects a shift from microscale inductive units toward tissue-scale spatial integration. Compared with isolated HFG models, composite structures better capture the spatial relationships among stratified epidermis, dermal matrix, and HF-like appendages, thereby providing an architectural basis for subsequent vascular, neural, immune, and MPS integration. Nevertheless, these models still primarily address structural integration rather than dynamic function and should not be equated with complete reconstruction of HF cycling, IP, or inflammatory responses. Skin–HF composite structures should therefore be viewed as an important intermediate stage in the progression from structurally oriented HF organoids toward functional HF-MPS.

## 3 Reconstructing immune competence in hair follicle models

In the context of HF-MPS construction, a truly immune-competent HF model should recapitulate key functional states of the native HF immune niche *in vitro*. These include the maintenance of immune homeostasis and IP, immune-cell regulation of hair cycling and post-injury regeneration, and inflammatory tissue damage following the breakdown of immune tolerance.

### 3.1 Immune homeostasis and immune privilege maintenance in normal hair follicles

Normal HFs rely on local immune homeostasis to restrain aberrant immune activation and autoimmune attack, with IP representing the most characteristic immune-protective mechanism. IP is thought to be mainly localized to the lower portion of anagen HFs, particularly the region surrounding the hair bulb. This state is maintained through restricted antigen presentation, local immunosuppressive signaling, and controlled immune-cell activation, thereby helping preserve HF structural integrity and regenerative potential (Shafiei-Jahani et al., 2026).

Within this regulatory network, regulatory T cells (Tregs) form an important cellular link between immune tolerance and the HFSC niche. Selective depletion of skin Tregs can induce T cell-mediated HF inflammation. Mechanistically, these cells suppress effector T-cell activation and expansion primarily by expressing the high-affinity IL-2 receptor and consuming IL-2, rather than through the CTLA-4 pathway (Cohen et al., 2024). These findings indicate that local Tregs are essential for maintaining immune privilege within the HFSC niche.

Antigen-presenting cells (APCs), particularly dendritic cells (DCs) and their epidermal subset, Langerhans cells (LCs), constitute a major local antigen-presenting system in the HF and help regulate the balance between immune tolerance and T cell-mediated inflammation (Bertolini et al., 2020). During IP maintenance, HF epithelial cells downregulate MHC-I/II expression, thereby reducing self-antigen presentation to effector T cells and limiting aberrant immune activation. In parallel, HF epithelial cells, melanocytes, and immune cells such as Tregs produce immunosuppressive mediators, including TGF-β, IL-10, and α-MSH, which suppress DC/LC maturation and inflammatory activation (Ito et al., 2004). Epithelial CD200 can also engage CD200 R on myeloid cells, including DCs, thereby restraining excessive activation (Rosenblum et al., 2004). Together, these mechanisms establish and sustain the IP state.

**FIGURE 2:**
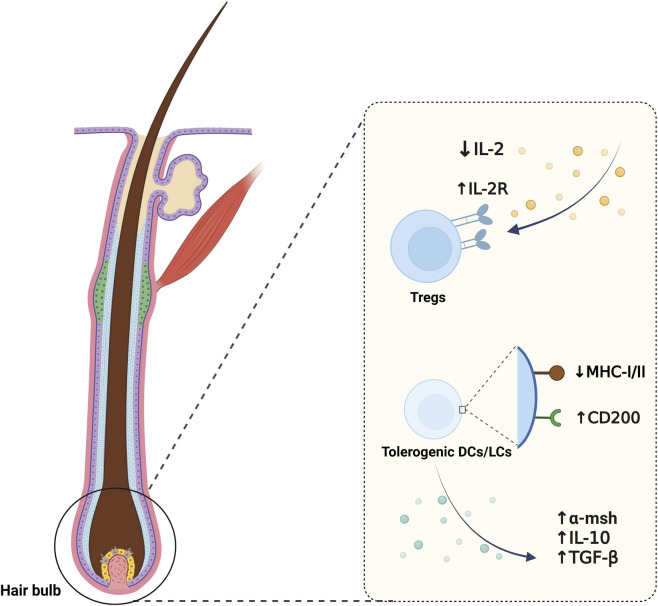
Cellular and molecular regulation of hair follicle immune privilege.

Accordingly, IP reconstruction in immune-competent HF-MPS should not be defined by the mere incorporation of Tregs, DCs, LCs, or other immune cells into the culture system. Rather, it should be demonstrated by measurable restriction of antigen presentation, preservation of immunosuppressive signaling, and tunable responses to inflammatory stimulation. Figure 2 summarizes the cellular and molecular mechanisms underlying HF IP maintenance.

### 3.2 Immune-cell regulation of hair follicle cycling and regeneration

Beyond maintaining local immune homeostasis, immune cells are increasingly recognized as functional regulators of the HFSC niche. Hair cycling has traditionally been attributed mainly to epithelial–mesenchymal interactions and canonical pathways such as Wnt/β-catenin, BMP, and SHH (Ouspenskaia et al., 2016). However, recent studies indicate that immune cells can also contribute to HFSC activation, differentiation, and hair-cycle transitions, thereby functioning as active components of the HFSC niche.

Macrophages are among the most representative immunoregulatory cells in the HF microenvironment, and their functional states vary with hair-cycle stage, tissue injury, and local inflammatory context (Huang et al., 2024). During the telogen-to-anagen transition, skin-resident macrophages undergo apoptosis accompanied by changes in Wnt-related gene expression. Induced macrophage apoptosis can promote premature HFSC exit from quiescence, whereas pharmacological blockade of Wnt ligand release delays hair growth (Castellana et al., 2014). These findings suggest that macrophage apoptosis and associated Wnt signaling may contribute to cyclic HFSC activation and anagen entry. Macrophages may also participate in catagen progression and telogen maintenance through stage-specific inhibitory cues such as FGF-5, although these roles remain context-dependent and require further validation (Gao et al., 2021; Kiselev and Park, 2024; Zhang and Chen, 2024).

In injury-associated regeneration, macrophages act as dynamic regulators linking inflammatory activation to tissue repair (Guenin-Mace et al., 2023). In full-thickness wound and hair-plucking models, inflammatory M1-like macrophages activate Lgr5^+^ HFSCs and promote injury-induced HF regeneration through the TNF-TNFR1-AKT/β-catenin axis. Notably, both TNF deficiency and excessive TNF expression impair *de novo* HF formation, indicating that macrophage-derived inflammatory signalling regulates regeneration in a dose-dependent manner rather than acting as a purely damaging signal (Wang et al., 2017). As repair proceeds, macrophages typically shift from an early pro-inflammatory state involved in debris clearance and inflammatory initiation toward a later reparative state that supports angiogenesis, inflammation resolution, and ECM remodelling through factors such as VEGF, IGF-1, IL-10, and TGF-β (Rajendran et al., 2020; Hassanshahi et al., 2022; Xu et al., 2024; Hsieh et al., 2025). This phenotypic plasticity creates a supportive immune microenvironment for anagen initiation and post-injury regeneration.

Tregs also participate in hair-cycle regulation. Skin-resident Tregs express high levels of the Notch ligand Jagged1 and regulate HF epithelial stem cell differentiation through the Jagged1-Notch axis (Ali et al., 2017). Thus, Tregs are not merely immunosuppressive cells; they also function as niche-associated regulators that directly influence HFSC behaviour and hair-cycle dynamics.

**FIGURE 3:**
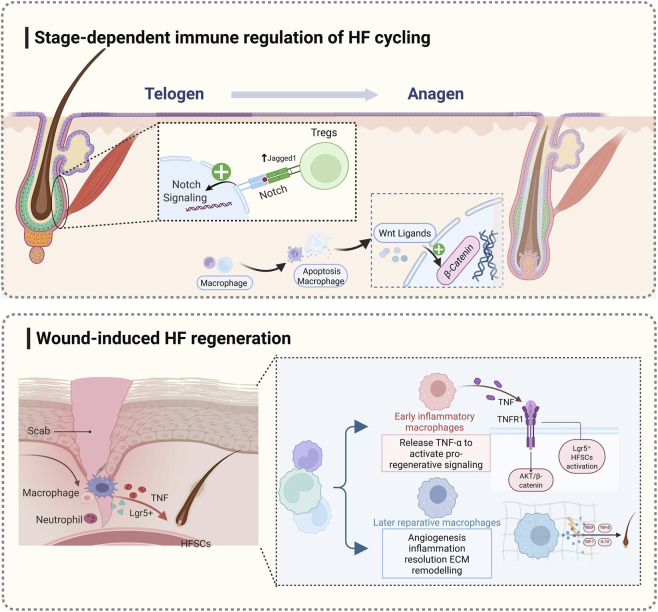
Immune regulation of hair follicle cycling and regeneration.

Together, these findings indicate that hair-cycle transitions and post-injury regeneration are not governed solely by epithelial–mesenchymal interactions. Local immune cells also regulate HFSC activation, differentiation, and the regenerative microenvironment. For immune-competent HF-MPS, model evaluation should therefore move beyond immune-cell survival or marker expression. It should assess whether incorporated immune cells exert functional effects on hair-cycle transitions, HFSC activation, and key pathways such as Wnt/β-catenin and Jagged1-Notch signaling. Strategies for achieving these functions through co-culture, dynamic perfusion, or immune-signal delivery are discussed in subsequent sections. Figure 3 illustrates how macrophages, Tregs, and local immune signals participate in HF cycling, HFSC activation, and post-injury regeneration.

### 3.3 Immune dysregulation and pathological HF destruction

Under pathological conditions, the local HF immune network can shift from homeostatic control to inflammatory amplification, ultimately contributing to structural and functional damage. This dysregulation manifests differently across immune-mediated alopecias. In non-scarring alopecia such as AA, the central pathological event is IP collapse, characterized by MHC-I/II upregulation, activation of the IFN-γ/JAK–STAT inflammatory axis, enhanced antigen presentation, and CD8^+^ T-cell-mediated HF attack (Bertolini et al., 2020; Zhao et al., 2025). By contrast, scarring alopecias such as LPP and FFA are marked by chronic immune-inflammatory damage to the bulge-region HFSC niche, accompanied by fibrotic remodeling and irreversible loss of HF architecture (Harries et al., 2018; Senna et al., 2022). These disease patterns indicate that HF immune dysregulation is not limited to transient inflammation, but can compromise HFSC niche stability, hair-cycle maintenance, and tissue integrity.

Neuro-immune-vascular interactions further shape the perifollicular inflammatory microenvironment. Dense sensory nerve fibers and microvascular networks surrounding HFs influence immune-cell recruitment and regulate local vascular responses and inflammatory intensity through neuropeptide signaling. Substance P (SP) is associated with mast cell activation, increased vascular permeability, and neurogenic inflammation (Marek-Jozefowicz et al., 2023). In contrast, calcitonin gene-related peptide (CGRP) is not simply a pro-inflammatory mediator; during acute injury, it can also contribute to tissue repair by modulating neutrophil and macrophage responses (Lu et al., 2024). Thus, HF immunopathology is not driven by a single immune-cell population, but reflects a dynamic imbalance shaped by immune, neural, vascular, and matrix microenvironments.

Accordingly, immune-competent HF-MPS should capture not only normal immune homeostasis and immune-cell-mediated regenerative regulation, but also the transition from immune protection to inflammatory injury. Ideally, these models should reproduce key events such as IP collapse, IFN-γ/JAK-STAT activation, effector T-cell involvement, and HFSC niche damage under controlled and measurable conditions. Such functional readouts are necessary for developing HF-MPS as disease-relevant platforms for mechanistic studies and drug evaluation in immune-mediated alopecias.

## 4 Engineering immune-competent HF-MPS: from local immune control to system-level integration

### 4.1 Local immune microenvironment engineering: spatial and temporal control of HF immune states

Under physiological conditions, HF immune regulation is both regionally and temporally organized. Distinct HF compartments exhibit different immune features: the lower anagen follicle, particularly the bulb region, is closely associated with IP maintenance, whereas the bulge region and HFSC niche are linked to local immune protection and stem cell homeostasis. Meanwhile, local immune signals change dynamically during hair-cycle transitions, injury repair, and inflammatory responses. In conventional organoid culture, the simple addition of cytokines or random co-culture of immune cells is insufficient to recapitulate region-specific immune-cell recruitment, activation, interaction, and temporal dynamics within the HF.

Accordingly, the key to immunologically functionalizing HF organoids is not to increase immune components indiscriminately, but to position immune signals at the appropriate location and time. The ECM-based delivery strategies discussed in Section 2 highlight the importance of local signal control for HFG maturation. Similar material-engineering principles may be extended to immunoregulatory cues to model IP maintenance, inflammatory perturbation, and recovery.

At present, studies directly targeting regional IP reconstruction in HF organoids through local immune delivery remain limited. Nevertheless, related work in immune engineering provides useful conceptual and technical references. Multicomponent injectable hydrogels, for example, can locally deliver peptide antigens and induce antigen-specific Treg responses, suggesting that hydrogels may serve as platforms for constructing local tolerogenic immune microenvironments (Verbeke et al., 2017). Although not HF-specific, this approach suggests that hydrogel-based systems may help shape Treg-biased immune responses and inform future attempts to regulate IP-related immune tolerance in HF-MPS. In addition, contact-dependent signals such as Jagged1-Notch are difficult to reproduce by adding soluble factors to the bulk culture medium alone. Immobilizing Jagged1-mimetic ligands within porous HA hydrogel scaffolds can activate Notch signalling through material-surface ligand presentation and modulate local macrophage recruitment and polarization (Deng et al., 2022).

During inflammation resolution and repair, local delivery of immunomodulatory exosomes may also help shape the immune microenvironment. M2-derived exosomes can induce the transition of M1 macrophages toward an M2-like phenotype, and their encapsulation within hydrogel scaffolds enables sustained release, thereby creating a locally pro-reparative immune microenvironment (Kim et al., 2019). Unlike material-based delivery, photobiomodulation (PBM) may be better suited for temporally controlled immune modulation. PBM has been shown to promote M2-like macrophage polarization, suggesting that optical modulation could serve as a tool for stage-specific regulation of immune-cell states (Zhang et al., 2024).

Although most of these studies were not conducted in HF-specific models, they collectively suggest that local delivery, ligand presentation, sustained exosome release, and temporally controlled intervention may provide useful engineering references for constructing controllable local immune states in HF organoids. Future studies should determine whether these strategies can be adapted to model HF-specific immune processes, including IP maintenance, IP collapse, and post-inflammatory recovery.

### 4.2 HF-MPS integration strategies: overcoming static co-culture bottlenecks

Although engineering the local immune microenvironment can improve the spatial and temporal precision of immune-signal regulation, local modulation alone is insufficient to sustain long-term, dynamic multicellular co-culture. Immune-competent HF models must maintain the functional states of epithelial cells, DPCs, HFSCs, and diverse immune-cell populations within the same system, despite their differing requirements for culture medium, growth factors, oxygen supply, and metabolic conditions (Papp et al., 2024). Under static co-culture conditions, the requirements of 1 cell type may compromise the function of another, thereby limiting model stability. Conventional organoids are also constrained by oxygen and nutrient diffusion; as construct size increases, internal hypoxia, metabolic waste accumulation, and necrotic core formation become more likely (Wörsdörfer and Ergün, 2021; Zhao et al., 2021). This challenge is further amplified in immune-competent HF models, where HF-like structure formation requires prolonged culture, whereas primary immune cells are prone to apoptosis, exhaustion, or phenotypic drift *in vitro* (Lee et al., 2020; Bogoslowski et al., 2023).

Static culture alone cannot resolve these challenges. MPS provide a more systematic route for integrating multicellular architecture, dynamic exchange, and functional regulation (Sahoo et al., 2026). In this context, HF-MPS refer to *in vitro* systems that build on HF organoids by incorporating engineering modules such as compartmentalized culture, ALI, dynamic perfusion, and vascularization. Their value lies in enabling dynamic mass exchange, spatial separation of multicellular microenvironments, and temporally controlled input of immune signals and immune cells, thereby overcoming key limitations of static culture in long-term maintenance and functional control.

#### 4.2.1 Compartmentalized culture: spatially resolving multicellular compatibility

Compartmentalized culture refers to the establishment of multiple, partially separated culture chambers within the same *in vitro* system, while permitting signal exchange through diffusion, microchannels, or perfusion (Kook et al., 2017). Its purpose is to provide distinct local microenvironments for different cell types or tissue regions without eliminating intercellular communication. Several existing platforms offer useful design references. Compartmentalized microfluidic perfusion systems with porous barriers can support the co-culture of different cell types in adjacent chambers while maintaining inter-chamber fluid exchange and cell-cell crosstalk (Ramadan et al., 2020). Similarly, dual-chamber epithelial organ-on-chip systems connected by microchannels have been used to model compartmentalized epithelial–stromal interactions (Tantengco et al., 2021). Although these studies were not designed for HF organoids, they provide useful principles for multicellular co-culture in HF-MPS. In parallel, 3D printing and mold-based strategies have enabled spatial organization of epidermal, dermal matrix, and HF-like units within skin-HF composite structures (Abaci et al., 2018; Kang et al., 2022).

For immune-competent HF-MPS, compartmentalized culture should also provide a spatial basis for immune-cell input and migration. Modeling immune-cell extravasation and migration generally requires multicompartment systems that include endothelial channels, ECM hydrogel regions, and epithelial barrier regions, together with perfusion and chemotactic gradients to guide immune cells into tissue compartments (Van Os et al., 2023a).

#### 4.2.2 Air–liquid interface culture: establishing a mature epithelial–skin context

ALI is primarily used to promote epithelial maturation (Hong et al., 2023). By reproducing the physiological configuration in which the basal surface remains in contact with liquid medium while the apical surface is exposed to air, ALI culture promotes keratinocyte terminal differentiation and lipid barrier formation (Lee et al., 2023; Kim et al., 2024). Across epithelial models, ALI culture has been shown to generate tissues that more closely resemble their physiological counterparts (Tian et al., 2023; Shirai et al., 2024). In skin models, ALI more effectively supports keratinocyte differentiation, keratinization, and barrier formation than submerged culture (SM), thereby improving the physiological relevance of *in vitro* skin equivalents (Fauzi et al., 2020). In HF-MPS construction, ALI may therefore help establish more mature epithelial organization and skin tissue stratification, providing a structural basis for subsequent immune-cell incorporation.

#### 4.2.3 Dynamic perfusion and vascularized interfaces: from mass exchange to immune-cell trafficking

Conventional static culture relies mainly on passive diffusion for the exchange of oxygen, nutrients, and metabolic waste. Dynamic perfusion, by contrast, can improve nutrient supply, oxygen delivery, and waste removal through continuous or periodic fluid exchange, thereby enhancing the long-term maintenance of complex skin-HF models. The skin and hair-on-a-chip model developed by Ataç and colleagues suggests that dynamic perfusion can support the *ex vivo* maintenance of skin and hair-associated tissues, providing an experimental basis for dynamic culture of skin-HF systems (Ataç et al., 2013). Pumpless skin-on-chip platforms further indicate that dynamic culture can be achieved through relatively simple microfluidic designs and can help maintain tissue architecture and culture stability in full-thickness skin models (Mohamadali et al., 2023).

For immune-competent HF models, dynamic perfusion may also enable stage-specific delivery of immune cells, inflammatory mediators, or therapeutic agents (Van Os et al., 2023a). This controlled input could help address the timescale mismatch between HF maturation and the limited *in vitro* lifespan of primary immune cells. In a lung inflammation organ-on-chip model, immune cells were dynamically introduced through microfluidic perfusion and migrated across an endothelial barrier into inflamed tissue regions under chemotactic gradients (Van Os et al., 2023b). Thus, dynamic perfusion not only improves mass exchange but also provides an engineering basis for temporally controlled immune-cell input (Campisi et al., 2022).

However, dynamic perfusion alone is insufficient to reproduce the full process of immune-cell entry into tissues. *In vivo*, immune cells typically enter the interstitium through vascular endothelium via rolling, adhesion, transendothelial migration, and local recruitment (Gonlugur and Efeoglu, 2004; Schober and Weber, 2005). Therefore, immune-competent HF-MPS require not only perfusion, but also endothelialized vascular-like channels that can provide both a transport route and a physiologically relevant immune-cell entry interface.

Current vascularization strategies include self-assembled vascular-like networks, endothelialized perfusable channels, 3D-printed hierarchical vascular networks, and microfluidic vascularized MPS. These approaches aim to generate vascular structures through endothelial self-organization, endothelial lining of preformed lumens, additive manufacturing of multiscale branching networks, or integration of perfusable vascular units into organ-on-chip systems. Although they can improve mass exchange and provide controllable vascular interfaces, their application to immune-competent HF-MPS remains constrained by lumen maturity, long-term perfusability, faithful modeling of immune-cell transendothelial migration, and insufficient HF-specific validation (Hong et al., 2023; Shiwarski et al., 2025).

For immune-competent HF models, endothelialized perfusable channels and microfluidic vascularized MPS are particularly relevant because they can provide perfusion, an endothelial barrier, and an immune-cell input interface within the same system. In skin equivalents, endothelial cell-lined perfusable vascular channels have been shown to support nutrient delivery and molecular transport (Mori et al., 2017). Self-assembled and perfusable microvasculature-on-chip platforms can further model leukocyte transport and migration within microvascular networks (Hirth et al., 2024). More closely related to immune-competent skin modeling, a vascularized immune-competent skin-on-chip has integrated a stratified epidermis, dermis-like matrix, and endothelialized microvascular network. In this system, HSV infection induced IL-8 production, and perfused human neutrophils adhered to the endothelium, extravasated across the vascular barrier, and migrated toward the infected epidermis (Sun et al., 2022).

Although these studies are highly relevant to HF-MPS design, current evidence remains largely based on skin MPS, generic microvascular chips, or immune-cell migration models. Direct validation of HF-specific vascular–immune interactions is still lacking. Future HF-MPS designed to model AA-like inflammation, IP collapse, or post-inflammatory recovery will need to demonstrate that vascularized interfaces can remain compatible with DPCs, HFSCs, and immune cells over time, while enabling immune cells to enter perifollicular tissues in a manner that more closely resembles *in vivo* recruitment.

#### 4.2.4 Innervated interfaces: a prospective module for neuro–immune–vascular crosstalk

Innervation may serve as a forward-looking module for further functionalizing HF-MPS. Cutaneous sensory nerves regulate vascular responses, immune-cell activity, and tissue repair through neuropeptides such as SP and CGRP (Jiang et al., 2024; Lu et al., 2024). Their value, therefore, extends beyond the addition of sensory function; they may provide a regulatory interface for neuro-immune-vascular crosstalk.

Hair-bearing skin organoids have been shown to contain sensory neurons and Schwann cell-like components, suggesting that skin organoids have the intrinsic potential to incorporate neural elements (Lee et al., 2020). More instructive examples come from innervated epidermal chip models. A microfluidic sensory neuron-keratinocyte co-culture system combined with a ramped ALI configuration enabled the formation of a three-dimensional innervated epidermis-like structure with functional neuro-epidermal connections (Ahn et al., 2023). Similarly, an MEA-compatible microfluidic model using iPSC-derived sensory neurons and keratinocytes has been used to assess neuron–keratinocyte interactions through electrophysiological readouts, providing a technical reference for future functional evaluation of HF-MPS innervation (Bessy et al., 2026). Overall, innervation is currently best viewed as a prospective module for HF-MPS. Its roles in hair cycling, IP maintenance, and inflammatory alopecias remain biologically plausible but still require HF-specific validation.

### 4.3 Dynamic monitoring and digital interpretation: from endpoint assays to quantitative readouts

As HF-MPS increasingly integrate local immune regulation, dynamic perfusion, vascularized interfaces, and innervated interfaces, their evaluation can no longer rely solely on endpoint staining or single-marker assays (Shrimali et al., 2025). Processes such as IP maintenance, IP collapse, immune-cell migration, inflammatory injury, and pharmacological responses are inherently dynamic. Model assessment, therefore, needs to move from static endpoint observation toward continuous, multiparametric monitoring.

Metabolic and microenvironmental sensing can first be used to determine whether the model remains in a stable culture state. Parameters such as oxygen, pH, lactate, glucose, and metabolic waste products reflect perfusion efficiency, metabolic stress, and long-term culture stability. General MPS studies have incorporated multi-analyte sensing and integrated sensors for real-time monitoring of metabolites, including oxygen and lactate (Dornhof et al., 2022; Izadifar et al., 2024). In parallel, microsensors and imaging-based approaches are increasingly being explored to monitor immune-cell behavior (Kumar et al., 2024; Morais et al., 2024). Fluorescent nanosensors integrated into microfluidic systems, for example, have enabled real-time monitoring of macrophage immune responses at the population level (Cho et al., 2021). Intravital imaging has also allowed dynamic tracking of immune-cell behavior in living tissues (Mukhopadhyay, 2022). These sensing strategies provide useful technical references for HF-MPS stability assessment, but still need to be adapted to HF architecture and immune-functional readouts.

As HF-MPS acquire immune-functional features, they will also generate continuous, multidimensional datasets. AI and computational models may therefore support the processing and interpretation of dynamic feedback from HF-MPS (Wang et al., 2023). P system models have been used to simulate T-cell behavior and immune-cell interactions (Ciobanu, 2005), while VirtualCytometry can infer immune-cell differentiation states from single-cell RNA sequencing data (Kim et al., 2020). In addition, microfluidic platforms combined with morphology-based deep learning have enabled automated recognition of neurotoxicity-associated morphological changes in MPS (Han et al., 2024). These studies suggest possible routes for digitalizing HF-MPS evaluation. However, HF-MPS-specific training datasets, evaluation criteria, and validation frameworks remain to be established.

### 4.4 Evidence boundaries and failure modes

Although the strategies discussed above provide feasible routes toward immune-competent HF-MPS, they should not be interpreted as evidence that the native HF immune niche has already been fully reconstructed. Current support remains uneven: perfusion and vascularized interfaces are relatively close to skin- or hair-related models, whereas innervation, integrated sensing, and AI-assisted interpretation are still largely extrapolated from other MPS platforms. Moreover, immune-cell incorporation may introduce instability, including excessive inflammatory activation, impaired HF-like structure maintenance, immune-cell apoptosis, exhaustion, or phenotypic drift. Future HF-MPS should therefore be evaluated not by short-term cell survival or marker expression alone, but by whether they can maintain, perturb, and partially restore HF-specific immune functions. A comparison of current strategies, representative examples, and remaining limitations is provided in Table 2.

**TABLE 2:** Engineering strategies for constructing immune-competent HF-MPS.

Engineering module	Main challenge addressed	Core strategy	Relevance to immune-competent HF-MPS	References
Local immune microenvironment engineering	Global cytokine supplementation or random immune-cell co-culture cannot recapitulate regional immune regulation	Local delivery, ligand presentation, sustained exosome release, and temporally controlled intervention	Models IP maintenance, inflammatory perturbation, and post-inflammatory recovery, although most evidence remains non-HF-specific	Verbeke et al. (2017), Kim et al. (2019), Deng et al. (2022), Zhang et al. (2024)
Compartmentalized culture	Conflicting culture requirements among multiple cell types	Multichamber design, microchannels, porous barriers, and region-specific microenvironments	Provides distinct local conditions for epithelial cells, DPCs, HFSCs, and immune cells	Ramadan et al. (2020), Tantengco et al. (2021)
ALI culture	Insufficient epithelial maturation and immature skin tissue background	Apical air exposure with basal medium contact	Promotes epithelial maturation and barrier-like structure formation, providing a more mature tissue background for immune integration	Fauzi et al. (2020), Tian et al. (2023), Shirai et al. (2024)
Dynamic perfusion	Insufficient mass exchange in static culture and limited stage-specific immune-cell input	Continuous or periodic perfusion with temporally controlled delivery of factors, cells, or drugs	Improves oxygen and nutrient supply, waste removal, and helps address the timescale mismatch between HF maturation and immune-cell lifespan	Ataç et al. (2013), Van Os et al. (2023b)
Vascularized interface	Perfusion alone does not reproduce a physiological immune-transport interface	Endothelialized channels, self-assembled microvasculature, and vascularized MPS	Supports immune-cell adhesion, transendothelial migration, and recruitment into tissue compartments	Mori et al. (2017), Sun et al. (2022), Hirth et al. (2024)
Innervated interface	Lack of a regulatory interface for neuro–immune–vascular crosstalk	Sensory neuron–keratinocyte co-culture and microfluidic innervated epidermal models	Serves as a prospective module for modeling neurogenic inflammation and neuro–immune regulation	Ahn et al. (2023), Bessy et al. (2026)
Dynamic monitoring and digital interpretation	Endpoint staining cannot capture dynamic immune processes	Metabolic sensing, imaging-based tracking, and AI-assisted interpretation	Enables assessment of culture stability, immune-cell behavior, and multiparametric dynamic responses, but HF-specific training and validation remain insufficient	Cho et al. (2021), Dornhof et al. (2022), Izadifar et al. (2024)

## 5 Translational applications of immune-competent HF-MPS

The central value of immune-competent HF-MPS lies in providing human-relevant platforms for mechanistic studies and drug evaluation in immune-mediated HF diseases. Conventional animal models and static HF culture systems cannot simultaneously recapitulate human HF architecture, immune-cell migration, and dynamic pharmacological responses. As a result, they remain limited in dissecting processes such as IP collapse, chronic inflammatory damage, and HFSC niche injury (Castro et al., 2022; Zhao et al., 2023).

### 5.1 Human-relevant modelling of immune-mediated hair disorders

This need is particularly evident in AA. The core pathological features of AA include IP collapse, activation of the IFN-γ/JAK-STAT axis, enhanced antigen presentation, and CD8^+^ T-cell-mediated HF attack (Zhao et al., 2025). Because these immunopathological events are relatively well defined, AA represents a practical entry point for developing immune-competent HF-MPS. In AA-oriented HF-MPS, local immune perturbation could be induced using inflammatory stimuli such as IFN-γ or IL-15, or through the introduction of effector T cells. Major readouts may include MHC-I/II expression, JAK–STAT activation, inflammatory chemokine release, immune-cell infiltration, and HF-like structural damage (Papadopoulos et al., 2000). Such systems could help reconstruct early events of AA-like immune injury in a human-relevant local microenvironment, thereby providing disease-modelling information that is difficult to obtain from animal models or static HF cultures.

Compared with AA, modelling scarring alopecias such as LPP and FFA in HF-MPS is more challenging. These diseases involve not only local immune inflammation, but also HFSC niche injury, sebaceous gland abnormalities, ECM remodelling, and irreversible fibrosis over prolonged disease courses (Harries et al., 2018; Senna et al., 2022). Therefore, fully reconstructing such complex pathology in short-term HF-MPS is currently unrealistic. A more feasible role for HF-MPS is to serve as a mechanistic platform for dissecting specific links among chronic inflammatory signalling, macrophage-state transitions, ECM remodelling, and HFSC niche damage. In this sense, near-term LPP/FFA-oriented HF-MPS are better positioned to model defined pathological modules rather than to reproduce the complete natural history of disease.

### 5.2 Dynamic drug evaluation: from endpoint measurements to process-based readouts

Another important application of HF-MPS is dynamic drug evaluation. Conventional drug screening largely relies on endpoint staining, cell viability assays, or single-marker detection, making it difficult to capture the continuous transition from inflammatory suppression to structural recovery. By contrast, HF-MPS can enable longitudinal observation of inflammation induction, immune-cell input, pharmacological intervention, HF structural damage, and subsequent recovery under perfusion conditions, thereby providing multidimensional readouts that more closely approximate dynamic *in vivo* responses (Sabaté Del Río et al., 2023). Taking JAK inhibitors, first-line therapeutic agents for severe AA, as an example (Mao et al., 2023; Sinclair et al., 2025), HF-MPS could be used to evaluate next-generation JAK inhibitors or monoclonal antibodies targeting pathogenic cytokines. Through integrated sensing networks, such systems may continuously monitor local inflammatory mediators, such as IFN-γ, and generate dynamic response profiles within the HF microenvironment.

These real-time readouts may allow more precise assessment of pharmacodynamic and pharmacokinetic–pharmacodynamic (PK/PD) parameters, including onset of action, response duration, and tissue penetration, thereby informing dose optimization and treatment-window design. However, HF-MPS should be positioned as human-relevant *in vitro* platforms for comparative pharmacodynamic assessment and mechanistic validation, rather than as substitutes for clinical efficacy evaluation.

### 5.3 Patient-derived HF-MPS for personalized disease modeling and drug evaluation

Patient-derived MPS (P-MPS) provide a potential platform for individualized studies of HF diseases (Ko et al., 2024). In principle, integrating patient-derived keratinocytes, DPCs, iPSCs, and peripheral immune cells could generate HF-MPS that more closely reflect individual genetic backgrounds, immune states, and HF microenvironmental features (Lee et al., 2020; Hong et al., 2023). For heterogeneous alopecias such as AA, LPP, and FFA, patient-derived HF-MPS may enable comparative analysis of interindividual differences in IP collapse, inflammatory amplification, HFSC niche damage, and drug responses (Bertolini et al., 2020; Senna et al., 2022).

However, patient-derived HF-MPS should currently be positioned as tools for individualized mechanistic investigation and exploratory drug-response assessment, rather than as mature platforms for clinical decision-making. Key limitations include the decline of DPC inductive capacity after *in vitro* expansion, the long differentiation timelines and batch-to-batch variability of iPSC-derived systems, immune-cell apoptosis, exhaustion, or phenotypic drift during long-term culture, and insufficient standardization of multicellular co-culture and clinical efficacy validation (Higgins et al., 2013; Bogoslowski et al., 2023).

### 5.4 HF-MPS in regenerative medicine: toward functional evaluation of engineered HF grafts

In regenerative medicine, the near-term value of HF-MPS is not to provide clinically transplantable HFs directly, but to serve as platforms for functional evaluation and quality control of engineered HF-like structures. Previous advances in HFG assembly, ECM-mediated niche regulation, and skin–HF composite construction have laid the foundation for HF regeneration (Nakao et al., 2007). With the further integration of dynamic perfusion, vascularization, and immune evaluation, HF-MPS may help assess DPC inductivity, HFSC states, hair shaft-like structure formation, vascular and neural interface integration, and potential immune risks (Xu et al., 2022; Zhou et al., 2025).

However, engineered HF donor banks remain a long-term goal. Current limitations include insufficient long-term maintenance of hair cycling, unstable control of hair shaft growth direction and tissue polarity, immature vascular and neural integration, and limited *in vivo* validation of post-transplantation growth capacity and immune compatibility (Abreu and Marques, 2022; Hong et al., 2023). Therefore, the realistic role of HF-MPS in regenerative medicine is to optimize HF construction conditions and support pre-transplantation quality assessment, rather than to directly replace existing donor sources for hair transplantation.
